# Homologous Muscle Contraction during Unilateral Movement Does Not Show a Dominant Effect on Leg Representation of the Ipsilateral Primary Motor Cortex

**DOI:** 10.1371/journal.pone.0072231

**Published:** 2013-08-21

**Authors:** Shin-Yi Chiou, Ray-Yau Wang, Kwong-Kum Liao, Yea-Ru Yang

**Affiliations:** 1 Department of Physical Therapy and Assistive Technology, National Yang-Ming University, Taipei, Taiwan; 2 Department of Neurology, Taipei Veterans General Hospital, Taipei, Taiwan; 3 Faculty of Medicine, National Yang-Ming University, Taipei, Taiwan; 4 Taipei City Hospital, Taipei, Taiwan; Brigham and Women’s Hospital, Harvard Medical School, United States of America

## Abstract

Co-activation of homo- and heterotopic representations in the primary motor cortex (M1) ipsilateral to a unilateral motor task has been observed in neuroimaging studies. Further analysis showed that the ipsilateral M1 is involved in motor execution along with the contralateral M1 in humans. Additionally, transcranial magnetic stimulation (TMS) studies have revealed that the size of the co-activation in the ipsilateral M1 has a muscle-dominant effect in the upper limbs, with a prominent decline of inhibition within the ipsilateral M1 occurring when a homologous muscle contracts. However, the homologous muscle-dominant effect in the ipsilateral M1 is less clear in the lower limbs. The present study investigates the response of corticospinal output and intracortical inhibition in the leg representation of the ipsilateral M1 during a unilateral motor task, with homo- or heterogeneous muscles. We assessed functional changes within the ipsilateral M1 and in corticospinal outputs associated with different contracting muscles in 15 right-handed healthy subjects. Motor tasks were performed with the right-side limb, including movements of the upper and lower limbs. TMS paradigms were measured, consisting of short-interval intracortical inhibition (SICI) and recruitment curves (RCs) of motor evoked potentials (MEPs) in the right M1, and responses were recorded from the left rectus femoris (RF) and left tibialis anterior (TA) muscles. TMS results showed that significant declines in SICI and prominent increases in MEPs of the left TA and left RF during unilateral movements. Cortical activations were associated with the muscles contracting during the movements. The present data demonstrate that activation of the ipsilateral M1 on leg representation could be increased during unilateral movement. However, no homologous muscle-dominant effect was evident in the leg muscles. The results may reflect that functional coupling of bilateral leg muscles is a reciprocal movement.

## Introduction

It is widely thought that unilateral hand movements are associated not only with activation of the contralateral primary motor cortex (M1) but also with the co-activation of the M1 ipsilateral to the movement, based on data from functional magnetic resonance imaging studies [1]–[5]. Such studies also found that the ipsilateral M1 is involved in the processing of unilateral movements of the upper limbs [5], [6]. Additionally, dynamic fluctuations in ipsilateral M1 activity were correlated with contralateral M1 responses [4], suggesting that there may be some interactions between bilateral M1s during unilateral movements of the upper limbs. This possibility is supported by transcranial magnetic stimulation (TMS) studies, which showed that the amount of interhemispheric inhibition (IHI) changed during unilateral movements of the upper limbs [7], [8]. In addition, reductions in short-interval intracortical inhibition (SICI) and corticospinal outputs were compatible with changes in IHI [8], [9]. These findings confirmed the existence of interactions between bilateral M1s during unilateral movements in the upper limbs.

In our previous work, co-activation of the ipsilateral M1 was observed during unilateral ankle dorsiflexion, and the activities of contralateral and ipsilateral M1s were relevant to the task, according to the results of an independent component analysis [6]. This finding indicates that the ipsilateral M1 is involved in the motor execution of lower limbs with the contralateral M1, and the mechanism of the ipsilateral M1 co-activation occurs at a cortical level, which is similar with the findings of the upper limbs. Similarly, the effect of muscle strengthening on knee extensors was transferable to the opposite, untrained leg, and cortical plasticity in the untrained hemisphere was detected, as measured by intracortical disinhibition and an increase in corticospinal output [10]. However, this finding could not clarify whether a homologous muscle-dominant effect occurs in leg representation, similar to the phenomenon of hand representation, as the authors only tested one leg muscle contralateral to the training leg. It is difficult to infer the effects of the specific responses on leg representations according to the findings of the upper limbs, since neurophysiological responses between upper and lower limbs are distinct. For instance, the H-reflexes in lower limb muscles were more effective when conditioning contractions were performed in a proximal muscle compared to a distal one; however, these reflexes did not show an effective dependency on upper limb muscles [11], [12]. This finding can be explained by the fact that lower limb muscles are involved in postural adjustments. Moreover, reciprocal voluntary movement is a functional movement of the lower limbs, whereas bimanual voluntary movements are easy to perform in a mirror direction, compatible within a homologous muscle-dominant effect on hand representation of the ipsilateral M1. Therefore, muscles that could induce a dominant change in the excitability of the ipsilateral M1 on leg representation may differ from that on arm/hand representation. The present study investigates the responses of TMS paradigms, SICI and corticospinal outputs, on leg representation of the ipsilateral M1 during unilateral movements with homo- or heterologous muscles. Two lower limb muscles with different locations were recruited to clarify whether different effects were evident in proximal and distal muscles. The unilateral movements of the shoulder, elbow, knee, and leg as responses of motor evoked potentials (MEPs) and SICI were varied according to homologous muscles and the role of each muscle in functional movement synergies [7], [13].

The purpose of the present study was to investigate the effects of unilateral motor tasks on the excitability of the ipsilateral M1 on leg representation. We investigated whether the excitability of the ipsilateral M1 on leg representation could be changed dominantly by a muscle contracting in a unilateral movement and whether corticospinal output and intracortical activation mediate the muscle-dominant effect.

## Materials and Methods

### Subjects

Fifteen right-handed healthy volunteers (eight male and seven female) with an average age of 25.8 years [standard deviation (SD): 1.42 years] participated in the study and gave their written informed consent before participation. The experimental procedures were approved by the Institutional Review Board, Taipei Veterans General Hospital and were performed according to the ethical standards of the Declaration of Helsinki.

### Experimental Design

The subjects reclined on the examination bed in a semi-seated position with the hip flexing at 100°. Pillows were placed below the knees and behind the back to support the torso fully. Subjects were asked to relax both legs and to keep the electromyographic (EMG) signal silent. This was defined as the ‘rest’ condition. The subjects were then asked to activate their task muscles for forceful isometric contraction while keeping the EMG of the left target muscles silent. This was defined as the ‘active’ condition. Task muscles were set on the right side and included the anterior deltoid (AD), flexor carpi radialis (FCR), rectus femoris (RF), and tibialis anterior (TA) muscles. Target muscles were determined on the left lower limb contralateral to the task muscles and included the rectus femoris (c-RF) and tibialis anterior (c-TA) muscles. Thus, there were four pairs of active conditions for each target muscle: 1) AD contraction (AD task) with c-RF/c-TA relaxation; 2) FCR contraction (FCR task) with c-RF/c-TA relaxation; 3) RF contraction (RF task) with c-RF/c-TA relaxation; and 4) TA contraction (TA task) with c-RF/c-TA relaxation. For the AD task, the initial position was set at 30° shoulder flexion. Subjects lifted their right arm to 90° shoulder flexion with the elbow extended and forearm pronated. For the FCR task, subjects flexed the wrist to the end range of motion with 90° elbow flexion and the forearm and wrist in a neural position on the pillow. For the RF task, subjects extended the right knee from slight flexion to a straight position. For the TA task, subjects dorsiflexed the right ankle from slight plantarflexion to full dorsiflexion. Four active conditions were applied in a randomized order subsequent to the rest condition. A muscle trigger stimulus technique was used to start the cortical stimulation. The subject’s maximal EMG activity (EMGmax) was first recorded while performing a maximal voluntary contraction of each target muscle. The peak amplitude of rectified EMGmax was recorded, and 75% of the EMGmax was set as the muscle trigger level. When the EMG signal reached the trigger level, the TMS stimulus was initiated. The instruction to the subjects was, “When you are ready, initially contract your muscle on the right side to reach the trigger line and completely relax the muscle on left side”. Additionally, the subject was requested to keep contracting around 1 s after hearing the sound of the TMS. The inter-stimulus interval between EMG onset and TMS stimulation was set at 100 ms for receiving the optimal facilitating effect [14]. TMS was applied on right M1 during the active condition. The EMG activity of the target muscle (c-RF or c-TA) was displayed on the screen to provide feedback to both the participant and the experimenter. For individual traces, EMG activity on target muscles was recorded for a total of 400 ms with 140 ms prior to the onset of the TMS stimulus. Trials in which the activity of the target muscles exceeded 25 µV of background noise were excluded from analysis [9]. All measurements were collected on the same day.

### Electromyographic Recording

Surface electrodes were positioned on the skin overlying both target and task muscles, with an active lead on the muscle bellies and a reference lead 4 cm below the active lead. The ground lead was placed on the left forearm. The EMG signals were amplified with filters set at 20 Hz to 3 kHz, and recorded on a computer (Neuropack MEB-9100; Nihon Kohden Corp., Tokyo, Japan).

### TMS Measurements

TMS was applied on the right M1 through a double cone coil (110-mm coil diameter) connected to two Magstim 200 stimulators via a BiStim module (The Magstim Company Limited, Spring Gardens, Whitland, Carmarthenshire, UK). A swim cap was put on the subject’s head so that ink marks regarding the coil position could be drawn, allowing re-positioning of the coil throughout the experiments. TMS was placed 1–2 cm posterior from vertex and slightly rotated to obtain an optimal position for induction of the largest MEP response in the c-RF/c-TA muscles at a given intensity. Measures of motor cortical excitability included resting motor threshold (RMT), MEP recruitment curves (RCs), and SICI in the right M1. All paradigms were applied on c-RF and c-TA separately. Because of the length of the physiological measurements and to avoid excessive fatigue, all measurements were completed in three to four sessions.

### Recruitment Curves of MEPs

RCs were measured in the left target muscles when the right task muscles were either at rest or at active conditions in all subjects. Stimulus intensities started at the RMT, defined as the lowest intensity of TMS output required to evoke MEPs of at least 50 µV in the peak-to-peak amplitude in at least three of five consecutive trials [15], and were increased gradually at 0.2 times the RMT. The average RMTs of c-RF and c-TA were 62.50±6.72% maximal output (range: 45∼70) and 57.07±8.92% maximal output (range: 55∼70), respectively. Less than one-half of the subjects had a stimulus intensity at 1.8 times the RMT, which was still below the 100% maximal output (four in c-RF; six in c-TA). Thus, a total of four different stimulus intensities were applied finally (1.0, 1.2, 1.4, and 1.6 times the RMT). According to previous reports [8], [16], a mean of five recorded MEPs resulted in good-to-high reliability in amplitude measures when a single hotspot technique was applied. Five MEPs were recorded at each stimulus intensity, and each TMS pulse was given every 5 s [8]. Several periods for rest were given to subjects between trials to avoid muscle fatigue. To normalize the individual MEP amplitudes, peripheral motor responses were measured. In the c-TA, a maximal motor response (M-max) was collected by stimulating the tibial nerve (1 ms rectangular pulse) with supramaximal intensity using bipolar surface electrodes placed around the fibular head. In the c-RF, a technique of femoral nerve magnetic stimulation (FNMS) with a double 70-mm coil was used to assess peripheral motor responses [17], [18]. Subjects lay supine on the examination bed with the left knee flexed at 90°. The intensity of the stimulus was set at 100% of maximal output of the Magstim 200 stimulator. The coil was placed above the femoral triangle just lateral to the femoral artery. An optimal location was then determined by identifying the position giving the greatest peak-to-peak amplitude in the c-RF after stimulation with single pulses.

### Short-interval Intracortical Inhibition

SICI in the right M1 was measured in the c-RF and c-TA, when the task muscles were either at rest or at active conditions in all subjects. The paradigm of SICI was similar to that described by Kujirai etal. [19], with a subthreshold conditioning stimulus followed by a suprathreshold test stimulus. The conditioning stimulus was set at an intensity of 80% RMT. This low-intensity stimulus does not activate corticospinal fibers and does not produce changes in the excitability of spinal motoneurons. The intensity for the conditioning stimulus was applied at rest condition and during active conditions consistently. The test stimulus was set to produce a control MEP of 0.3∼0.5 mV at rest and adjusted to match the control MEP during active conditions. Test stimuli were delivered 2.0 ms after conditioning stimuli. Five paired-pulses stimuli were applied with a 5-s inter-trial interval between two trials. Several periods for rest were given to the subjects between trials to avoid muscle fatigue.

### Data Analysis

An average prestimulus EMG activity was obtained by calculating the root mean square for a 40-ms prestimulus interval in each condition and intensity. The magnitude of the MEP was measured as the peak-to-peak amplitude and normalized with respect to the amplitude of the M-response. The mean ± standard error (SE) was used to present values of MEP RCs at both rest and active conditions. To determine the inhibitory effect on SICI, the peak-to-peak amplitude of each MEP was measured and, without normalizing with the M-response, all amplitudes were averaged for each stimulus condition. The amplitude of the conditioned MEP was expressed as a percentage of the mean unconditioned MEP amplitude, and the values were presented as mean ± standard deviation (SD).

### Statistics

The data from the c-RF and c-TA were analyzed separately. Two-way repeated-measures ANOVA was used to determine the effect of CONDITION (rest, AD task, FCR task, RF task, and TA task) and stimulus INTENSITY (1.0, 1.2, 1.4, and 1.6 times the RMT) on MEP RCs and on background EMG activity. Further, repeated-measures ANOVA was applied to determine the effect of CONDITION on SICI. A post hoc simple contrast test with the first reference category was used following the analysis of the main effect. Then, the values of each active condition for the MEP amplitude and SICI were examined by one-way ANOVA following a Tukey’s post hoc test for multiple comparisons. Significance was set at *p*<0.05.

## Results

### Cortical Activation of the Ipsilateral Motor Cortex during Active Conditions by Contracting Muscles on the Right Side

**Figure 1:**
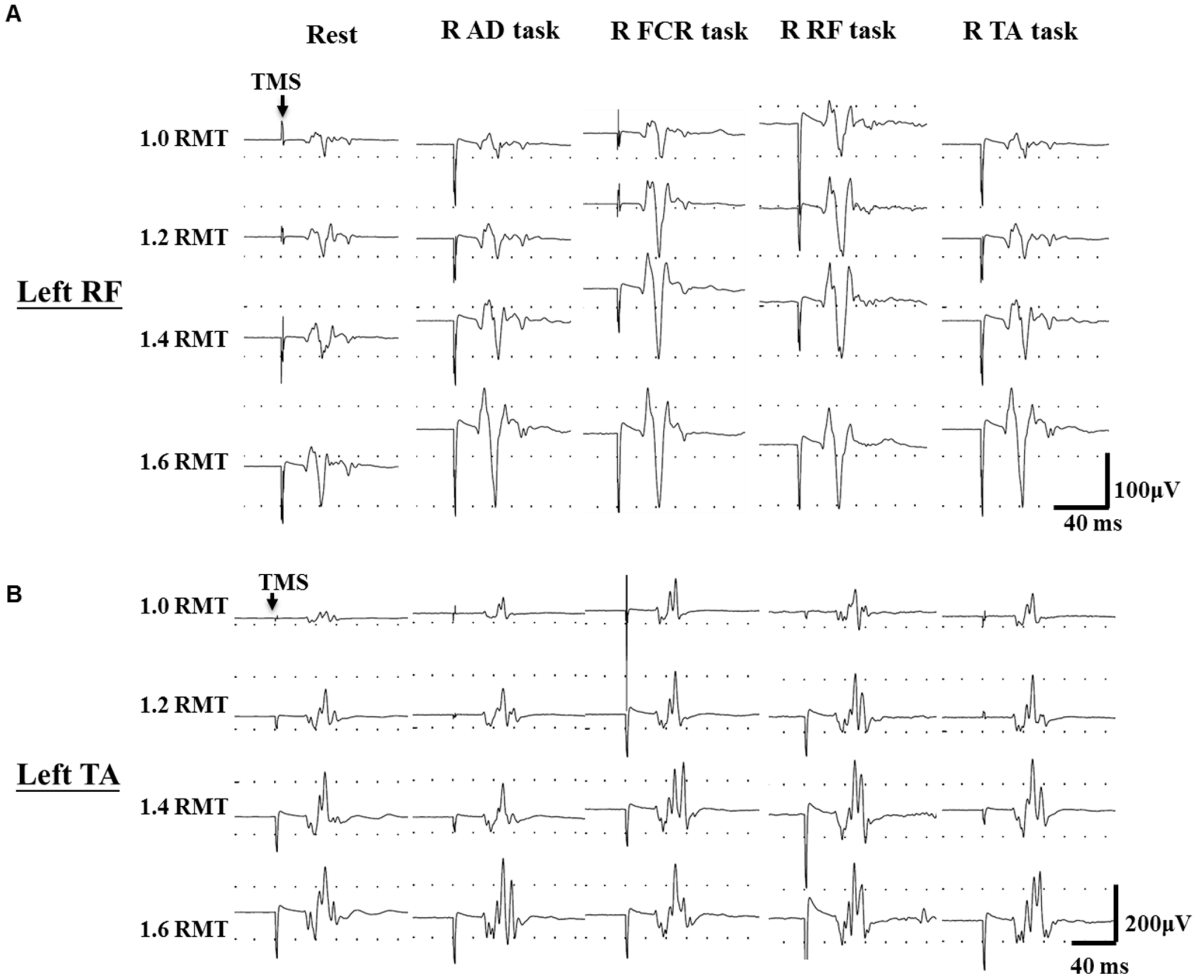
Typical recording of recruitment curves. Recording of motor evoked potentials from the left rectus femoris (RF) and left tibialis anterior (TA) muscles on a representative subject during different motor tasks of right side limbs. Arrows indicate delivery of transcranial magnetic stimulation (TMS). RMT: resting motor threshold; R: right; AD: anterior deltoid; FCR: flexor carpi radialis; RF: rectus femoris; TA: tibialis anterior.

**Figure 2:**
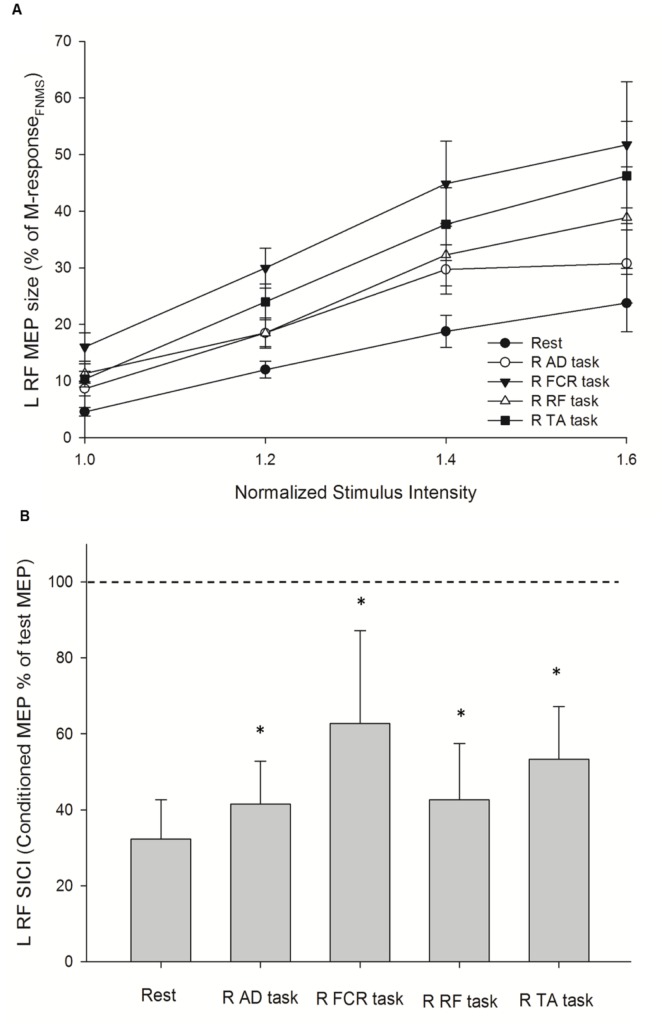
Increased corticospinal output and decreased intracortical inhibition of the left rectus femoris (L RF) muscle during unilateral motor task. (A) Recruitment curves of motor evoked potential (MEP) at rest and during four active conditions that were performed by muscles on the right side. The abscissa shows intensity of transcranial magnetic stimulus expressed relative to the resting motor threshold in each subject. The ordinate shows MEP amplitudes as a percentage of the M-responses collected via femoral nerve magnetic stimulation (M-response_FNMS_). Data are presented as the mean ± standard error from all 15 subjects. (B) Ratio of short-interval intracortical inhibition (SICI) at rest and during four active conditions. The size of the conditioned MEP is expressed as a percentage of the amplitude of the test MEP (horizontal dotted line). Data are presented as the mean ± standard deviation from all 15 subjects. Asterisks indicate statistically significant differences from the rest condition (*p<0.05) by repeated-measures ANOVA following a post hoc contrast test. R: right; AD: anterior deltoid; FCR: flexor carpi radialis; RF: rectus femoris; TA: tibialis anterior.

Figure 1 illustrates the left RF and left TA MEPs recorded in a single subject while performing different motor tasks with the right-side limb. Responses of MEP RCs and SICI on the left RF and left TA were examined separately. Repeated-measures ANOVA showed that there was an effect of INTENSITY (F_1.24, 16.07_ = 14.58; *p* = 0.004), and CONDITION (F_4, 52_ = 21.22; *p*<0.001), and their interaction CONDITION × INTENSITY (F_4.08, 53.00_ = 4.99; *p = *0.008) on MEP RCs of the left RF (Figure 2A). A post hoc contrast test showed that MEP amplitudes at 1.2 times (*p* = 0.001), 1.4 times (*p* = 0.004), and 1.6 times (*p* = 0.003) the intensity were greater than that at 1.0 times the intensity. Additionally, the post hoc contrast test revealed a significant increase in MEP amplitude in the AD task (*p = *0.006), FCR task (*p*<0.001), RF task (*p = *0.003), and TA task (*p = *0.001) compared with the rest condition on the left RF at each intensity. For the analysis of SICI, repeated-measures ANOVA showed significant effects of CONDITION on SICI of the left RF (F = 38.40; *p*<0.001, Figure 2B). The post hoc contrast test revealed a significant attenuation of SICI at the AD task (41.54±11.28%; *p<*0.001), FCR task (59.02±27.56%; *p<*0.001), RF task (42.68±14.76%; *p<*0.001), and TA task (50.34±17.73%; *p*<0.001) compared with that at the rest condition (30.54±12.10%) on the left RF.

**Figure 3:**
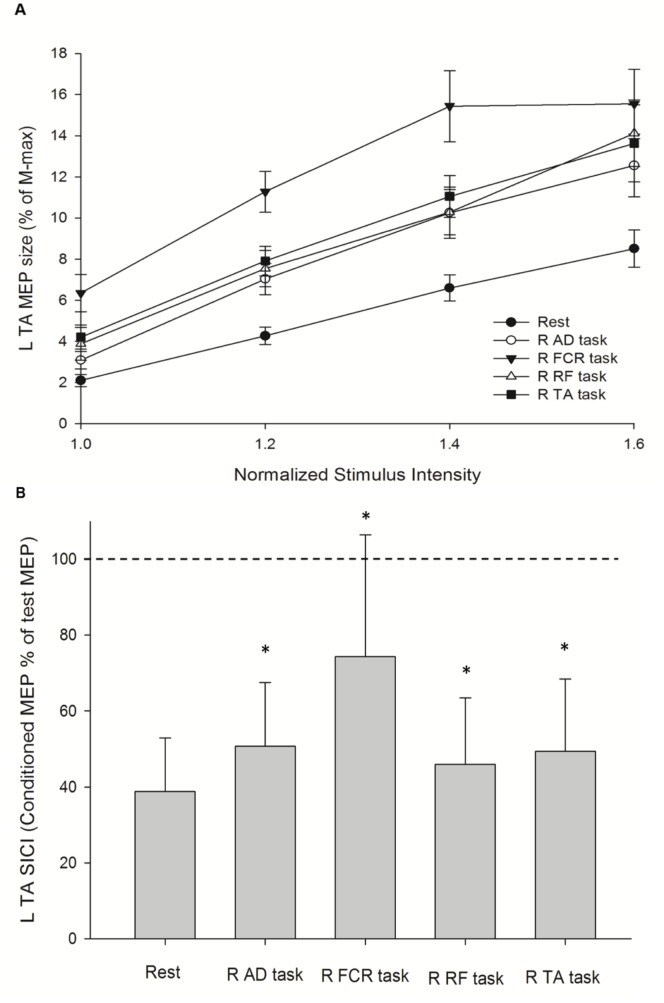
Increased corticospinal output and decreased intracortical inhibition of the left tibialis anterior (L TA) muscle during unilateral motor task. (A) Recruitment curves of motor evoked potential (MEP) at rest and during the three active conditions that were performed by muscles on the right side. The abscissa shows intensity of transcranial magnetic stimulus expressed relative to the resting motor threshold in each subject. The ordinate shows MEP amplitudes as a percentage of the L TA M-max. Data are presented as the mean ± standard error from all 15 subjects. (B) Ratio of short-interval intracortical inhibition (SICI) at rest and during the four active conditions. The size of the conditioned MEP is expressed as a percentage of the amplitude of the test MEP (horizontal dotted line). Data are presented as the mean ± standard deviation from all 15 subjects. Asterisks indicate statistically significant differences from the rest condition (*p<0.05) by repeated-measures ANOVA following a post hoc contrast test. R: right; AD: anterior deltoid; FCR: flexor carpi radialis; RF: rectus femoris; TA: tibialis anterior.

Similar steps were applied for analyzing the results of MEP RCs and SICI on the left TA at rest and during four active conditions. Repeated-measures ANOVA showed that there was effect of INTENSITY (F_1.04, 9.32_ = 41.87; *p*<0.001), and CONDITION (F_1.72, 15.44_ = 29.22; *p*<0.001), and their interaction CONDITION × INTENSITY (F_2.81, 25.29_ = 3.60; *p = *0.011) on MEP RCs of the left TA (Figure 3A). A post hoc contrast test showed that MEP amplitudes at 1.2 times, 1.4 times, and 1.6 times the intensity (*p*<0.001 for all) were greater than that at 1.0 times the intensity. Further, the post hoc contrast test revealed a significant increase in MEP amplitude at AD task (*p = *0.001), FCR task (*p*<0.001), RF task (*p*<0.001), and TA task (*p*<0.001) compared with the rest condition on the left TA at each intensity. For analysis of SICI, repeated measures ANOVA showed significant effects of CONDITION on SICI of the left TA (F_1.59, 22.24_ = 15.30; *p*<0.001, Figure 3B). SICI was decreased at the AD task (50.67±16.88%; *p*<0.015), FCR task (74.36±32.06%; *p*<0.001), RF task (46.01±17.47%; *p = *0.015), and TA task (49.41±18.97%; *p = *0.004) compared with the rest condition (38.85±14.08%) on the left TA.

### Cortical Activation of the Ipsilateral Motor Cortex during Homologous or Heterogeneous Muscle Contraction of the Right Side

To understand the effects of the interaction between CONDITION (active) and INTENSITY, the values of MEP amplitudes and SICI during active conditions were examined at each stimulus intensity. The results of one-way ANOVA showed an effect of active conditions on 1.0 and 1.2 times the MEP amplitudes of the left RF (1.0: F_3, 56_ = 3.13, *p = *0.033; 1.2: F_3, 56_ = 3.41, *p = *0.024; Table 1). A post hoc test was applied for between-conditions comparisons. At 1.0 times the MEP amplitudes, the FCR task significantly facilitated MEP amplitudes on the left RF compared with the AD task (*p = *0.025). At 1.2 times the MEP amplitudes, a significant increase in MEP amplitude at the FCR task compared with the AD task (*p = *0.04) and RF task (*p = *0.04) was observed. The results of one-way ANOVA also showed an effect of active conditions on SICI of the left RT (F_3, 56_ = 5.10; *p = *0.004). Post hoc comparisons showed that there was a significant decrease of SICI effect during the FCR task compared with the AD task (*p*<0.001) and RF task (*p = *0.001) on the left RF; however, during the RF task, the homologous muscle did not induce a prominent decrease of SICI effect on the left RF (Table 2).

**Table 1:** Amplitudes of motor evoked potential (% of M-response) during active conditions.

Intensity (Times of RMT)	Left RF				Left TA			
	R AD task	R FCR task	R RF task	R TA task	R AD task	R FCR task	R RF task	R TA task
1.0	8.65±1.26	16.01±2.50^a^	11.35±1.71	10.34±1.40	3.09±0.42	3.49±0.90^a^	3.11±0.80	2.26±0.58
1.2	18.47±2.38	29.96±3.51^a,b^	18.49±2.67	23.97±3.18	7.04±0.76	11.28±0.99^a,b,c^	7.55±0.88	7.91±0.72
1.4	29.70±4.36	44.86±7.53	32.32±5.50	36.14±3.05	10.26±1.25	15.44±1.73^a,b^	10.29±1.10	11.76±0.69
1.6	30.78±7.01	51.71±11.15	38.86±8.98	46.27±9.60	12.255±1.52	15.55±1.69	14.12±1.62	13.62±1.88

Data are presented as the mean ± SEM.

a, b, and^ c^ indicate significant differences between the R FCR task and the R AD task, between the R FCR task and the R RF task, and between the R FCR task and the R TA task, respectively. Significance level was set at *P*<0.05. RMT: resting motor threshold. R: right; AD: anterior deltoid; FCR: flexor carpi radialis; RF: rectus femoris; TA: tibialis anterior.

**Table 2:** Short-interval intracortical inhibition (%, SICI) during active conditions.

	Left RF				Left TA			
	R AD task	R FCR task	R RF task	R TA task	R AD task	R FCR task	R RF task	R TA task
**SICI (%)**	41.54±11.28	62.69±24.50^a,b^	42.68±14.76	53.35±13.87	50.67±16.87	74.36±32.0^a,b,c^	46.01±17.47	49.41±18.97

Data are presented as the mean ± SD.

a, b, and^ c^ indicate significant differences between the R FCR task and the R AD task, between the R FCR task and the R RF task, and between the R FCR task and the R TA task, respectively. Significance level was set at *P*<0.05. R: right; AD: anterior deltoid; FCR: flexor carpi radialis; RF: rectus femoris; TA: tibialis anterior.

In the left TA, the results of one-way ANOVA showed an effect of active conditions on 1.0, 1.2, and 1.4 times the MEP amplitudes (1.0: F_3, 56_ = 3.93, *p = *0.013; 1.2: F_3, 56_ = 5.17, *p = *0.003; 1.4: F_3, 56_ = 3.62, *p = *0.01; Table 1). A post hoc test showed that at 1.0 times the MEP amplitudes, the FCR task significantly facilitated MEP amplitudes on the left TA compared with the AD task (*p = *0.009). At 1.2 times the MEP amplitudes, a significant increase in MEP amplitude at the FCR task compared with the AD task (*p = *0.004), RF task (*p = *0.015), and TA task (*p = *0.033) was observed. At 1.4 times the MEP amplitudes, the FCR task also showed a prominent facilitatory effect on MEP amplitudes compared with the AD task (*p = *0.033) and RF task (*p = *0.035). The results of one-way ANOVA also showed an effect of active conditions on SICI of the left TA (F_3, 56_ = 5.12; *p = *0.003) and post hoc comparisons showed that there was a significant decrease in SICI during the FCR task compared with the AD task (*p = *0.02), RF task (*p = *0.005), and TA task (*p = *0.01) on the left TA. No significant decrease in SICI during the TA task on the left TA was observed (Table 2).

### Background EMG Activity

Background EMG activity on the left RF and left TA were examined separately. For the left RF session, repeated-measures ANOVA demonstrated no significant main effect for CONDITION (F_1.23, 17.28_ = 2.93; *p* = 0.1), and INTENSITY (F_2.02, 28.26_ = 1.03; *p* = 0.37), and their interaction CONDITION × INTENSITY (F_2.63, 36.85_ = 1.87; *p* = 0.16). The same was true for the left TA session. There were no significant main effect for CONDITION (F_1.93, 27.07_ = 1.84; *p* = 0.18), and INTENSITY (F_2.58, 36.05_ = 2.53; *p* = 0.08), and their interaction CONDITION × INTENSITY (F_2.61, 36.60_ = 41.87; *p* = 0.09) on background EMG activity.

## Discussion

The main findings of the present study are (1) activation of leg representation in the ipsilateral M1 was enhanced via unilateral movement; (2) unilateral movements with different contracting muscles influenced the size of the activation in the ipsilateral M1 on leg representation; and (3) a specific enhancement was *not* evident on homotopic representation, which differed from the phenomenon observed in the upper limbs.

Although a number of studies have investigated the co-activation of the ipsilateral M1 and homologous muscle-dominant effects in the upper limbs, to our knowledge, the present study is the first to investigate responses of the ipsilateral M1 during contraction of homologous and heterologous muscles in the *lower* limbs and link the phenomenon to responses of SICI and corticospinal excitability. As the connection between inter- and intracortical interactions regarding the homologous muscle-dominant effects in the upper limb muscles have been investigated in our previous work [6], similar protocols were applied in the present study to make reasonable comparisons.

According to the results of the MEP RCs, the facilitatory effect on the M1 ipsilateral to the unilateral movement occurs consistently at a stimulus intensity from low to high, even at the threshold intensity. The increases in MEP RCs most likely reflect the excitability of the cortical circuitry, the corticospinal cells, and the spinal alpha motor neuron pool [20]. Here, the motor neuron pool is less relevant, because the background EMG activities were well controlled in both rest and active conditions. In addition, unilateral movement seems to induce a general facilitation in the ipsilateral M1, as TMS at threshold intensities preferentially evoked indirect waves trans-synaptically within the motor cortex [21]. Further, our results demonstrated that SICI in the ipsilateral M1 was suppressed in all active conditions compared to the rest condition. Thus, these findings indicate that a facilitatory effect on the ipsilateral M1 may occur at the cortical level.

During unilateral muscle contraction, different types of contralateral effects have been reported [9], [22]–[24]. A homologous muscle-dominant effect has been reported in the upper limbs [25]–[27], and this effect is the result of both intra- and inter-hemispheric interactions, as IHI from the contralateral M1 to the ipsilateral M1 and SICI in the ipsilateral M1 have the highest degree of suppression during homologous muscle contraction [6], [8]. In the present study, prominent changes in both intracortical activation and corticospinal outputs of the ipsilateral M1 occurred on right wrist flexion. In other words, the activation of leg representation could be enhanced by a specific muscle contracting in a unilateral movement. Our results further demonstrate that this muscle-dominant effect occurs cortically, as the significant CONDITION effect on MEP amplitudes included stimulus intensity at 1.0 times the RMT, and there was a significant CONDITION effect on SICI. Our findings echo previous results regarding functional changes in the M1 ipsilateral to unilateral movements. Perez and Cohen [8] showed that interactions between intracortical circuits mediating SICI and interhemispheric glutamatergic projections between M1s contribute to changes in corticospinal outputs to the resting hand during force generation by the opposite hand. Additionally, Hortobágyi et al. [28] and Goodwill et al. [10] reported that unilateral motor practice increases motor outputs not only in the trained but also in the untrained muscle in the opposite limb that is modulated by changes in interhemispheric inhibition.

Although the excitability of the ipsilateral M1 on leg representation could be increased by several muscles, there was still a muscle that induced dominant facilitation on leg representation. However, this dominant muscle was not the homologous muscle, as the most prominent increases in corticospinal outputs and decreases in SICI in the RF and TA representations did not occur during right knee extension or right ankle dorsiflexion. In muscles of the upper limbs, the homologous muscle-dominant effect could be mediated via callosum motor fibers connecting the M1s in the two hemispheres. Fibers crossing the corpus callosum between homotopic representations are denser than those between non-homotopic representations. However, this would not explain the results of this effect in the lower limbs. Functional connectivity is another consideration to explain the different phenomena observed in the lower limbs. Alternatively, out-of-phase movements are the most functional movements of the lower limbs, and reciprocal inhibition of the homologous muscle on the opposite side is necessary for executing smooth movements in the lower limbs. Therefore, the homologous muscle-dominant effect in the ipsilateral M1 may be disrupted in the lower limbs during relatively unusual unilateral movements.

A unilateral hand movement seems most likely to induce stronger excitability in the ipsilateral M1, and it could be related to inter-limb coordination. Diagonal coordination between the arm and leg has been reported in humans [29]–[32], and leg muscle responses or even step initiation could be associated with opposite arm movements [33], [34]. Moreover, during rhythmic activity of the upper limb muscles, the EMG and reflex activities of the lower limb muscles is thought to be modulated [31]. In the present study, the right arm and left leg are in functional orientation; therefore, the effect of arm-leg coordination might result in a specific enhancement of leg representation during wrist flexion. Nevertheless, this interpretation only explains the effect partially, because right shoulder flexion did not induce a similar enhancement of leg representation in the ipsilateral M1, as observed during right wrist flexion.

There are some limitations of the present study. Due to technical limitations, we did not directly stimulate the femoral nerve for the Mmax of the RF. Instead, FNMS was used. However, FNMS was not strong enough to evoke the Mmax of the RF and resulted in a much larger normalized MEP response in the RF compared to that in the TA. Since we did not compare the results of the RF and TA directly, problems caused by such a discrepancy should be limited. The other limitation is the potential effect of the spread of current to other regions. Since we used a cone coil and measured RCs in the present study, it is difficult to prevent current spreading to other regions, especially at high intensity. Currents that spread to other regions might produce collateral effects but might not be sufficient to alter the responses of the leg muscle.

The present results have clinical relevance to neurological conditions. As the co-activation of the ipsilateral M1 and muscle-dominant effects are relevant to intra- and interhemispheric interactions, it is possible that the results could yield diagnostic or therapeutic benefits in certain clinical conditions, such as in patients with stroke where the balance between two hemispheres are reduced.

## Conclusions

In conclusion, the present study shows that during unilateral movements, the function of inhibitory circuits in leg representation of the ipsilateral M1 is partially suppressed and can result in increases in corticospinal outputs, similar to findings in upper limbs. However, the activation of leg representations is not via a homologous muscle-dominant effect. The unilateral movements that dominantly increase excitability of the ipsilateral M1 on leg representation may be related to their functional coupling with the leg muscles.
